# P-1149. Short versus Long Duration of Antibiotic Therapy in Pediatric Intensive Care Unit Patients with Uncomplicated Community Acquired Pneumonia: Impact of an Institutional Guideline Update

**DOI:** 10.1093/ofid/ofae631.1335

**Published:** 2025-01-29

**Authors:** Irza Jan, Jerod Nagel, Alison C Tribble, Daniel Riggsbee, Karen N Davidge

**Affiliations:** University of Michigan, Ann Arbor, Michigan; Michigan Medicine, Ann Arbor, Michigan; C.S. Mott Children's Hospital, University of Michigan Health, Ann Arbor, MI; University of Michigan, Ann Arbor, Michigan; University of Michigan Health, Ann Arbor, Michigan

## Abstract

**Background:**

Community acquired pneumonia (CAP) is a significant cause of morbidity and antibiotic overutilization among pediatric patients. The 2011 Infectious Diseases Society of America (IDSA) guidelines recommend a treatment duration of 10 days for pediatric CAP but recognize that shorter courses may be appropriate in certain settings. In February 2022, based on newer literature and extrapolations from the adult population, Michigan Medicine updated its institutional guidelines to recommend an antibiotic duration of 5 days to treat uncomplicated CAP in hospitalized pediatric patients. However, there remains limited evidence to support a shorter duration of therapy for patients admitted to the pediatric intensive care unit (PICU).

Baseline characteristics of patients treated for uncomplicated CAP in the PICU at Michigan Medicine from November 2020 to December 2023

**Figure:**
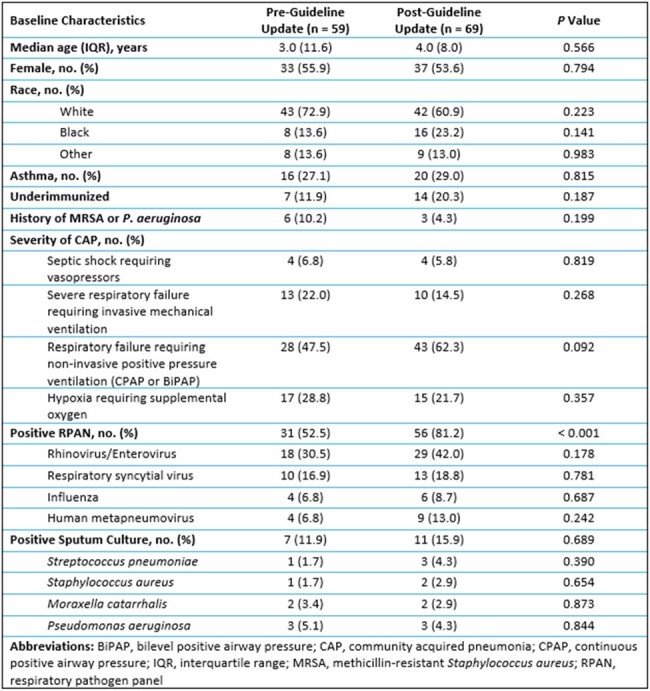

**Methods:**

This was a single-center, retrospective cohort study of patients aged 6 months to 17 years old with CAP who were admitted to the PICU at Michigan Medicine from November 2020 to December 2023. Patients with complicated CAP, hospital acquired or ventilator associated pneumonia, cystic fibrosis, severe neutropenia, solid organ or stem cell transplant, tracheostomy dependence, sickle cell disease, or suspicion for fungal infection were excluded from the study. The primary outcome was treatment failure, defined as a composite of readmission, emergency department or outpatient visit, need for antibiotic retreatment, or death attributable to CAP within 30 days of completing antibiotic therapy.

Clinical outcomes of patients treated for uncomplicated CAP in the PICU at Michigan Medicine before and after the institutional guideline update

**Figure:**
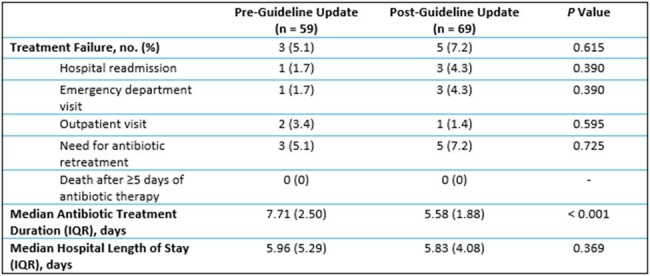

**Results:**

A total of 128 patients met eligibility criteria; 59 (46%) patients were treated before the guideline update and 69 (54%) were treated after the guideline update. The median antibiotic treatment duration was 7.71 days in the pre-guideline update group and 5.58 days in the post-guideline update group (*P* < 0.001). Three patients in the pre-guideline update group and five patients in the post-guideline update group experienced 30-day treatment failure, which was not a statistically significant difference (*P* = 0.615). There was no difference in the median hospital length of stay between groups.

**Conclusion:**

This study demonstrated that a shorter course of antibiotic therapy did not increase the rate of treatment failure compared to a standard course of therapy for patients admitted to the PICU with uncomplicated CAP.

**Disclosures:**

**All Authors**: No reported disclosures

